# DermAI 1.0: A Robust, Generalized, and Novel Attention-Enabled Ensemble-Based Transfer Learning Paradigm for Multiclass Classification of Skin Lesion Images

**DOI:** 10.3390/diagnostics13193159

**Published:** 2023-10-09

**Authors:** Prabhav Sanga, Jaskaran Singh, Arun Kumar Dubey, Narendra N. Khanna, John R. Laird, Gavino Faa, Inder M. Singh, Georgios Tsoulfas, Mannudeep K. Kalra, Jagjit S. Teji, Mustafa Al-Maini, Vijay Rathore, Vikas Agarwal, Puneet Ahluwalia, Mostafa M. Fouda, Luca Saba, Jasjit S. Suri

**Affiliations:** 1 Department of Information Technology, Bharati Vidyapeeth’s College of Engineering, New Delhi 110063, India; prabhav14.sanga@gmail.com (P.S.); arudubey@gmail.com (A.K.D.); 2 Global Biomedical Technologies, Inc., Roseville, CA 95661, USA; 3 Stroke Monitoring and Diagnostic Division, AtheroPoint™, Roseville, CA 95661, USAdrindersingh1@gmail.com (I.M.S.); rajvivs888@gmail.com (V.R.); 4 Department of Cardiology, Indraprastha Apollo Hospitals, New Delhi 110076, India; drnnkhanna@gmail.com; 5 Heart and Vascular Institute, Adventist Health St. Helena, St. Helena, CA 94574, USA; lairdjr@ah.org; 6 Department of Pathology, Azienda Ospedaliero Universitaria (A.O.U.), 09124 Cagliari, Italy; gavinofaa@gmail.com; 7 Department of Surgery, Aristoteleion University of Thessaloniki, 54124 Thessaloniki, Greece; tsoulfasg@gmail.com; 8 Department of Radiology, Massachusetts General Hospital, Boston, MA 02114, USA; mkalra@mgh.harvard.edu; 9 Department of Pediatrics, Ann and Robert H. Lurie Children’s Hospital of Chicago, Chicago, IL 60611, USA; jteji@mercy-chicago.org; 10 Allergy, Clinical Immunology and Rheumatology Institute, Toronto, ON L4Z 4C4, Canada; almaini@hotmail.com; 11 Department of Immunology, Sanjay Gandhi Postgraduate Institute of Medical Sciences, Lucknow 226014, India; vikasagr@sgpgi.ac.in; 12 Department of Uro Oncology, Medanta the Medicity, Gurugram 122001, India; puneet1923@gmail.com; 13 Department of Electrical and Computer Engineering, Idaho State University, Pocatello, ID 83209, USA; mfouda@isu.edu; 14 Department of Radiology, Azienda Ospedaliero Universitaria (A.O.U.), 09124 Cagliari, Italy; lucasabamd@gmail.com; 15 Department of Computer Science and Engineering, Graphic Era University (G.E.U.), Dehradun 248002, India

**Keywords:** skin lesions, attention, ensemble-based deep learning, reliability, validation

## Abstract

Skin lesion classification plays a crucial role in dermatology, aiding in the early detection, diagnosis, and management of life-threatening malignant lesions. However, standalone transfer learning (TL) models failed to deliver optimal performance. In this study, we present an attention-enabled ensemble-based deep learning technique, a powerful, novel, and generalized method for extracting features for the classification of skin lesions. This technique holds significant promise in enhancing diagnostic accuracy by using seven pre-trained TL models for classification. Six ensemble-based DL (EBDL) models were created using stacking, softmax voting, and weighted average techniques. Furthermore, we investigated the attention mechanism as an effective paradigm and created seven attention-enabled transfer learning (aeTL) models before branching out to construct three attention-enabled ensemble-based DL (aeEBDL) models to create a reliable, adaptive, and generalized paradigm. The mean accuracy of the TL models is 95.30%, and the use of an ensemble-based paradigm increased it by 4.22%, to 99.52%. The aeTL models’ performance was superior to the TL models in accuracy by 3.01%, and aeEBDL models outperformed aeTL models by 1.29%. Statistical tests show significant p-value and Kappa coefficient along with a 99.6% reliability index for the aeEBDL models. The approach is highly effective and generalized for the classification of skin lesions.

## 1. Introduction

An abnormality or a change in the appearance of the skin is referred to as a lesion [1,2,3]. In addition to moles, rashes, growths, discolorations, and other abnormalities, it can represent a variety of conditions. For several reasons, it is crucial to classify skin lesions [4,5,6,7,8]. A timely diagnosis and treatment of skin cancer depends on early detection and accurate classification of skin lesions. Detecting and treating skin cancer early can save lives. Secondly, healthcare professionals may have limitations in terms of accuracy and consistency when inspecting skin lesions manually [9,10,11]. To visually analyze skin lesions, dermatologists rely on their expertise and experience, but human error is common. By developing automatic classification systems using neural networks, we can enhance the accuracy and objectivity of skin lesion classification [12,13].

Convolutional Neural Networks (CNN) have been dominantly used in medical image classification and segmentation because of their property to automatically extract features that are different from a traditional neural network [14]. But for CNN models to perform, they require large training data, and thus, pre-trained models on large-scale datasets have been used as base models and fine-tuned for specific classification called transfer learning (TL) [15,16]. Various studies have been conducted on the use of pre-trained TL models for classification purposes [17,18,19,20,21], and they have performed well, but also these pre-trained models can have limitations like overfitting on a specific task [22,23]. Ensemble-based deep learning (EBDL) represents a significant improvement in the field of deep learning (DL), providing better performance than standalone models [24,25]. It enables the training of data of different types on different architectures to produce one single predictive output. To further enhance the performance of ensemble-based models, an attention mechanism is incorporated into the model architecture to increase its robustness and enable a more focused analysis of skin lesions.

We discussed the innovative application of aeTL models and aeEBDL approaches for skin lesion categorization. CNN models have traditionally been employed in medical image processing with promising results, although the requirement for large training datasets can be a disadvantage. To get around this, the work fixes them specifically for skin lesion classification by employing pre-trained models from large-scale datasets as foundation models. However, employing pre-trained models to overfit a certain job may have some downsides. The research introduces EBDL models, which mix different data kinds and architectures into a single predicted output to outperform solo TL models. Attention mechanisms are also included in the system for targeted classification of skin lesions. We designed various experiments for our study and achieved excellent performance on the models. We also preprocessed the dataset and balanced the classes to mitigate the bias in the models. Also, we performed statistical tests like the Wilcoxon p-test and Cohen’s Kappa for system reliability and robustness. Lastly, we performed a thorough reliability analysis, using misclassification of the models over the entire dataset and averaging the rate for each type of model. For the validation, we developed three hypotheses in this study based on the investigation of skin lesions. Our first hypothesis is that EBDL models will perform better than solo TL models due to their ability to capture various representations. Second, we expected that integrating an attention mechanism will improve the system’s performance over non-attention models. Attention models improve performance by allowing the model to focus on critical areas or skin lesion traits. Our final hypothesis is that attention-enabled EBDL (aeEBDL) models are superior to solo attention models due to the advantage of capturing multiple representations while focusing on salient elements.

In the spirit of our hypotheses, we developed seven TL models with *six* EBDL models using techniques like max voting, stack, and weighted average. An attention mechanism allows models to focus on salient features, thus improving accuracy. We embedded attention mechanisms into our TL models to envision seven new attention-enabled transfer learning (aeTL) models. Also, we incorporated three additional aeEBDL models. All of these models were evaluated on the HAM10000 dataset using five experimental protocols.

**Figure 1:**
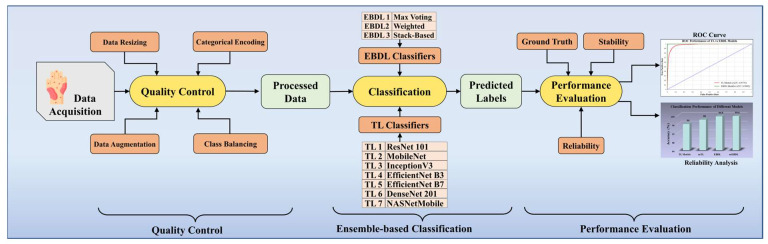
Online DermAI 1.0 of the proposed study. TL: transfer learning; EBDL: ensemble-based deep learning; and ROC: receiver operating characteristic.

Figure 1 portrays the online system of our scheme. With the data acquisition from the HAM10000 dataset, we perform quality control, including data resizing, categorical label encoding, data augmentation, and class balancing. The processed images are then classified using our classifier system, incorporating TL and EBDL classifiers. Finally, we enhance the robustness, reliability, and stability of our system by employing cross-validation protocols, receiver operating characteristics (ROC) curves, reliability analysis, and conducting statistical tests.

This paper follows a systematic flow, beginning with Section 2, which describes the literature review. Section 3 holds the methodology of our approach, including the dataset we have used, types of the classifiers, and the experimental protocols. Section 4 has the results of our performed experiments. Section 5 covers the performance evaluation of our proposed models, followed by Section 6 with a discussion of our approach and benchmarking of the models. Finally, the conclusions are drawn in Section 7.

## 2. Literature Review

Cancer is a disease that occurs through the uncontrolled multiplication of body cells, occupying peripheral tissues. Although skin cancer occurs less frequently than many other types of cancer, it is highly important due to its high mortality rate [26].

For a long time, machine learning (ML) algorithms like k-Nearest Neighbors (KNN) and Decision Trees (DT) have been widely used as supervised ML algorithms for image classification [27]. Sajid et al. [28] proposed a KNN-based classification system for skin lesions. The system incorporates filters to remove noise and extracts features from the lesion images. Their system successfully classifies the lesions into cancerous and non-cancerous categories. However, it is important to note that KNN systems are computationally expensive and do not perform well with high-dimensional inputs, which makes the algorithm expensive and unsuitable for skin lesion classification systems. Senan et al. [29] proposed a Support Vector Machine (SVM)-based system. They employed a Gaussian filter as part of the preprocessing process for image enhancement and utilized the active contour technique (ACT) to separate the lesion area from the healthy body. To extract features from the region of interest, they applied the Gray Level Co-occurrence Matrix (GLCM) method. While SVM can convert observations of possibly correlated variables into linearly uncorrelated variables using an orthogonal transformation, it may encounter difficulties in managing noisy input [30].

DL methods have enabled the development of intelligent medical-imaging-based diagnosis systems for medical image analysis. Lopez et al. [31] incorporated a VGG-net TL model for classifying skin lesions in their system. Serte et al. [32] proposed a deep CNN (DCNN) based on Gabor wavelets to detect malignant melanoma and seborrheic keratosis. In their method, an input image is decomposed into seven directional sub-bands, and eight parallel CNNs are used to generate probabilistic predictions based on these sub-band images and the input image. The sum rule is employed for decision fusion to classify the skin lesion.

Mirunalini et al. [33] developed an automated classification system using the InceptionV3 model to classify skin lesions as malignant or benign, and they also considered the cause of cancer for image classification. Mahbod et al. [34] proposed an automatic classification system using three pre-trained deep models: AlexNet, VGG16, and ResNet18, as deep feature generators. They then trained an SVM classifier using these features and obtained classification results by fusing the outputs. Younis et al. [35] developed a fast and reliable model using a pre-trained MobileNet, achieving good categorical accuracy over the HAM10000 dataset. Additionally, EBDL models have played a major role in improving detection accuracy. Harangiet et al. [36] proposed aggregating robust CNN models into one neural architecture, achieving classification using the weighted output of the member CNN models, outperforming all individual CNN models. Another ensemble-based model was introduced by Shehzal et al. [37], combining the abilities of EfficientNetV2S and Swin Transformer models to detect the early focal zone of skin cancer. This ensemble-based construction effectively minimized loss and increased the accuracy of the model.

After conducting a literature review, we concluded that there is a need for studies with superior performance analysis, more generalization, and reliability. Further validation is required for these AI models, as they have the potential to play a significant role in skin lesion detection.

## 3. Methodology

In this section, we will discuss the dataset and data demography, overall architecture, TL-based classification, EBDL paradigm, the use of attention in TL models and ensemble-based models, along with training methods, experimental protocol, and performance metrics.

### 3.1. Dataset and Data Demography

Dermatoscopy is a diagnostic technique that has shown promise in improving the diagnosis of pigmented skin lesions. The HAM10000 dataset, which stands for “Human Against Machine with 10,000 training images” [38], comprises a collection of 10,015 dermatoscopic images obtained from diverse Australian and Austrian patients using various imaging modalities. The data acquisition was carried out by two institutions: Cliff Rosendahl in Queensland, Australia, and the Medical University of Vienna, Austria. The dataset encompasses a representative range of important diagnostic categories related to pigmented skin lesions.

**Figure 2:**
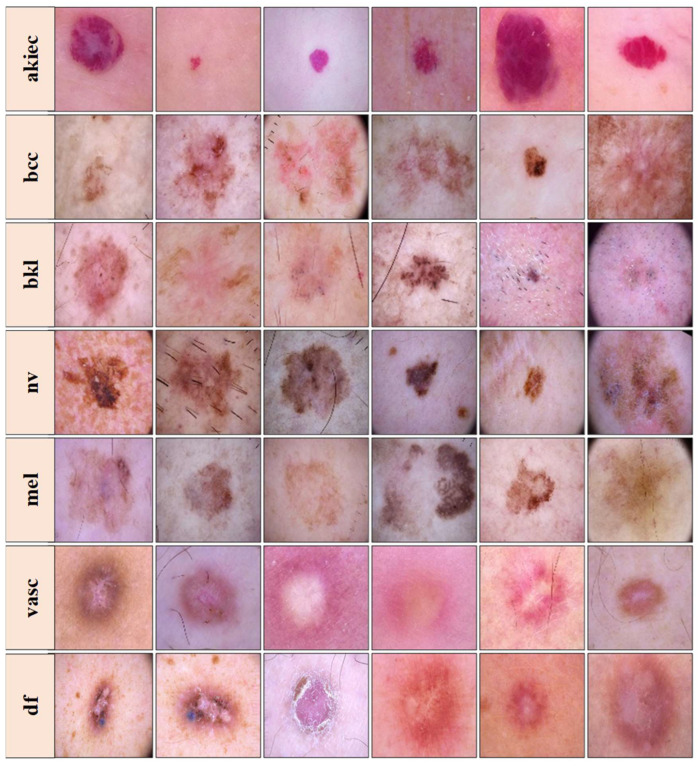
Images from the HAM10000 dataset. Top to bottom rows—Row 1: actinic keratoses (**akiec**), Row 2: basal cell carcinoma (**bcc**), Row 3: benign keratosis-like lesions (**bkl**), Row 4: melanocytic nevi (**nv**), Row 5: melanoma (**mel**), Row 6: vascular lesions (**vasc***)*, and Row 7: dermatofibroma (**df**), samples than others. This class imbalance could introduce bias during model training, favoring the majority classes over the minority ones.

The dataset visualized in Figure 2 is freely accessible for experimental purposes and serves as a valuable resource for research. It includes dermatoscopic images representing seven distinct classes: actinic keratoses and intraepithelial carcinoma/Bowen’s disease (**akiec**), basal cell carcinoma (**bcc**), benign keratosis-like lesions (solar lentigines/seborrheic keratoses and lichen-planus-like keratoses, **bkl**), dermatofibroma (**df**), melanoma (**mel**), melanocytic nevi (**nv**), and vascular lesions (angiomas, angiokeratomas, pyogenic granulomas, and hemorrhage, **vasc**). The ground truth for this dataset was established through pathology confirmation, master agreement, or confocal microscopy [39]. It is important to note that the images in the dataset may differ from what a layperson or end-user would provide in a real-world scenario. Images that were insufficiently magnified or out-of-focus were removed during the dataset curation process.

### 3.2. Quality Control

The dataset used for this project consists of images with an initial size of 450 × 600 pixels, which were later resized to 112 × 112 pixels to ensure uniformity for training a DL model. The dataset includes various classes, but there is an imbalance in the distribution of these classes, with some having significantly more samples than the other.

One specific class, “nv” (melanocytic nevi), had an excessive number of samples and outweighed other classes. To address this issue, a class balancing technique was implemented using data augmentation. Additional synthetic data were generated for the underrepresented class by augmenting the existing images. Various augmentation techniques were utilized, including cropping, image expansion, flipping (horizontal, vertical, and diagonal), color filtration, image shifting, and blurring. Some augmentations were also applied to the other classes but to a lesser extent.

Finally, the image labels were encoded using categorical encoding. The categorical class names were converted to single digits between zero and six, representing the classes efficiently for multi-classification purposes.

### 3.3. Global Architecture of the Proposed System

**Figure 3:**
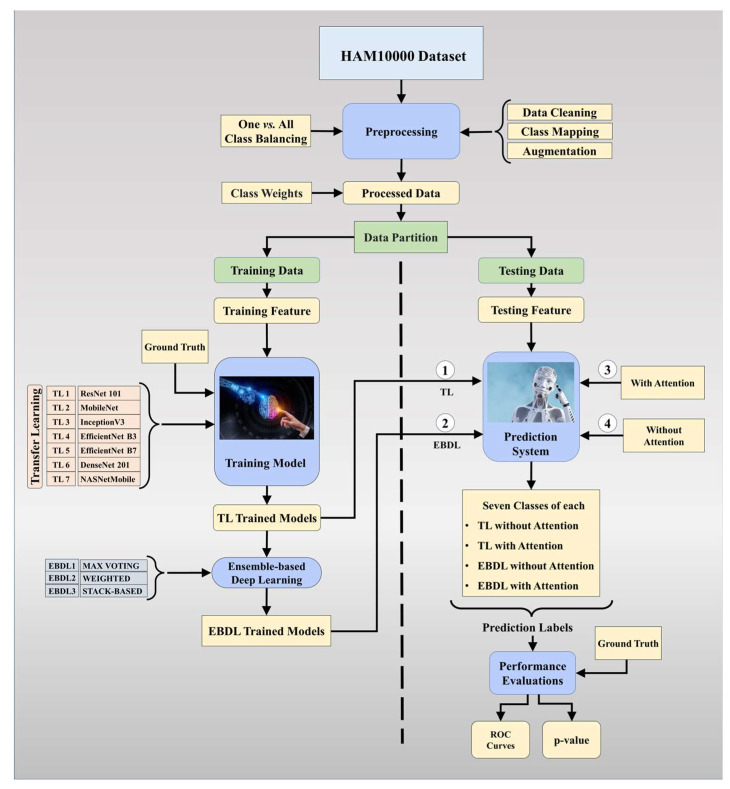
Global architecture of the proposed DermAI 1.0; TL: transfer learning; EBDL: ensemble-based deep learning; ROC: receiver operating characteristic.

The proposed overall architecture of DermAI 1.0 is depicted in Figure 3. In this system, we utilized and preprocessed the HAM10000 [38] dataset. We mapped meta-data to the corresponding images and ensured dataset cleanliness. The HAM10000 dataset contains 10,015 images, initially exhibiting an imbalance. To address this, we employed a one vs. all class balancing technique and augmented the dataset to increase the training data size for our models. Class weights were then applied to the processed data, enhancing the model’s sensitivity. The dataset was split into training and testing sets, with 80% allocated for training and 20% for testing purposes. The training data were used to train the tuned TL models and EBDL models. Subsequently, attention mechanisms were incorporated into these models to further improve accuracy and reliability.

In this study, we hypothesized that the mean accuracy of the EBDL models would surpass that of the individual TL models. Additionally, we anticipated that the inclusion of attention mechanisms would lead to better performance in the aeTL models compared to the fine-tuned TL models alone. Furthermore, we hypothesized that aeEBDL methods would outperform aeTL models. To evaluate the performance of the models, we conducted scientific validation as well as statistical analysis and calculated metrics such as precision, recall, F1-score, and AUC. These evaluations allow us to assess the effectiveness and reliability of our proposed system in detecting and classifying skin lesions accurately.

### 3.4. Architecture of the Classifiers

For building the skin lesion detection system, we utilized four different sets of classifiers: solo TL models, EBDL models, aeTL models, and aeEBDL models. All of these figures are visualized in Figure 4, Figure 5, Figure 6, Figure 7, Figure 8, Figure 9, Figure 10, Figure 11, Figure 12, Figure 13 and Figure 14.

#### 3.4.1. Solo Transfer Learning Models

TL [40] is considered a leading method for classification and offers several advantages over DL-based classification [17,41,42]. In our study, we utilized seven state-of-the-art TL models: ResNet101 [43], MobileNet [44], InceptionV3 [45], EfficientNet B3 [46], EfficientNet B7 [46], DenseNet-201 [47], and NASNet Mobile [48]. These models were pre-trained on the ImageNet dataset. By leveraging these advanced architectures as base models, we designed custom top layers for each model. These top layers consisted of a pair of dense layers with 4096 units, employing the ReLU activation function, followed by a dropout layer to mitigate overfitting. The final dense layer had 7 units, corresponding to the number of classes in our classification task. It utilized the softmax activation function to output probabilities for each class.

Using TL models like ResNet101, MobileNet, InceptionV3, EfficientNetB3, EfficientNetB7, DenseNet201, and NASNetMobile can be highly effective. These models allow us to leverage pre-trained networks that were initially trained on large datasets to learn important features from the data. ResNet is a renowned deep residual network that utilizes skip connections. ResNet101, with its 101 layers, is particularly well-suited for complex image recognition tasks, especially in multiclass classification scenarios. MobileNet, on the other hand, is designed to be lightweight and efficient, making it ideal for processing on mobile devices and low-resource systems. It employs depth-wise separable convolutions to reduce the number of parameters and computations while maintaining reasonable accuracy, which is advantageous for embedded systems in lesion classification. 

InceptionV3 incorporates multiple levels of feature extraction and boasts a fast-processing speed, outperforming other architectures in certain cases. EfficientNet provides excellent feature extraction and strikes a good balance between model size and accuracy. Among its architecture, EfficientNetB3 and EfficientNetB7 have shown remarkable performance in challenging image classification tasks. DenseNet, with its feature reuse and gradient flow mechanisms, is also notable. DenseNet201, with its 201 layers, is well-suited for complex image recognition tasks. Lastly, Neural Architecture Search Network (NASNet) is an architecture designed using neural architecture search methods, demonstrating excellent performance on mobile devices and resource-constrained environments. Given the unique characteristics of each model, it is highly beneficial to experiment with them individually to determine their performance on our specific task.

Ensemble-based techniques play a crucial role in medical imaging, helping to strengthen weak learners and improve overall performance and generalization. By combining multiple models, EBDL methods leverage the diversity of individual TL models. In our study, we utilized ensemble-based techniques to enhance the models’ capability to generalize. Each TL model had a different architecture, allowing them to learn distinct features from the data.

Among the EBDL methods we employed were softmax voting, weighted ensemble, and stack-based ensemble, with a specific focus on softmax voting. To create ensemble-based predictions using softmax voting, we collected predictions from each solo model and summed them along the prediction axis. The index of the highest value for each prediction determined the class with the majority votes, representing the ensemble prediction. By employing softmax voting, we effectively combined the predictions from multiple models, harnessing their collective knowledge.

#### 3.4.2. Ensemble-Based Transfer Learning Models

Additionally, we utilized the weighted average ensemble technique in our study. This method involved assigning weights to the models based on their significance in contributing to the ensemble prediction. The predictions from each model were averaged along the prediction axis, and the index of the highest value indicated the class with the highest weighted average ensemble prediction.

Furthermore, we incorporated a stack-based ensemble method for our EBDL models. This approach involved stacking the predictions from our models together to create a composite input, which was then used to train a meta-model. In our research, we used Logistic Regression as the meta-model to generate our final ensemble prediction. This process allowed us to further enhance the predictive power of our EBDL models.

#### 3.4.3. Attention-Enabled Transfer Learning Models

Attention mechanisms in deep neural networks are employed to improve model performance by enabling the network to selectively focus on crucial aspects of the input. In our study, we incorporated attention into our TL models to assess their impact on classification performance. As displayed in Equations (1)–(3). The attention weights were computed by considering the average prediction scores made with each model across the classes’ axis. Subsequently, self-attention was applied to the predictions of each model. By multiplying the attention weights element-wise with the predictions, we assigned greater importance to specific predictions based on their corresponding attention weights. This approach effectively enhances the model’s performance by enabling it to concentrate on relevant and significant features within the input.

#### 3.4.4. Attention-enabled Ensemble-based Deep Learning Models

In our study, we developed aeEBDL models by combining the predictions of multiple models and incorporating a self-attention layer on the concatenated prediction layer. The process of calculating attention weights involves considering the average prediction scores made using each model across the classes’ axis. As displayed in Equations (4)–(6) these attention weights are then multiplied element-wise with the predictions, assigning greater significance to specific predictions based on their corresponding attention weights.

(1) $${\;P}_{m}=[\;{\;P}_{m}\;\left(c_{1}\right),\;{\;P}_{m}\;\left(c_{2}\right)\;\ldots .\;{\;P}_{m}\;\left(c_{7}\right)]$$

(2) $${\;o}_{m}=[\;o_{m}\;\left(c_{1}\right),\;o_{m}\;\left(c_{2}\right)\;\ldots .\;o_{m}\;\left(c_{7}\right)]$$

(3) $${\;w}_{m}=\frac{\sum_{c=1}^{C}\;{\;P}_{m}\;\;\left(c\right)}{C}$$

(4) $${\;o}_{m}={\;P}_{m}{\;\times \;w}_{m}$$

(5) $$\bar{o\;}\left(c\right)=\frac{\sum_{m=1}^{M}\;o_{m}\left(c\;\right)}{M}\;\;$$

(6) $$P_{\mathrm{aeEBDL}}=\;\mathrm{argmax}\;[\;\bar{o\;}\;\left(c\right)\;]\;\;\;\;\;\;\;\;\;\;\;\;\;\;\;\;\forall \;c\;\mathrm{in}\;C$$

To generate ensemble predictions, we identified the class with the highest score from the attention-enabled combined prediction. This approach enhances the overall prediction performance by leveraging the attention weights to prioritize and emphasize certain predictions.

Here, ${\;P}_{m}$ denotes the m^th^ model’s prediction score, $o_{m}$ is the attention output of the m^th^ model, C denotes the number of classes in the multiclass framework, $w_{m}$ is the attention weights of the m^th^ model, M represents the total models, $\bar{o\;}\left(c\right)$ denotes the ensembled output from the solo M models, and $P_{\mathrm{aeEBDL}}$ is the final attention-enabled ensemble-based model output.

### 3.5. Training Parameters

We utilized the Idaho State University (ISU) GPU cluster to execute all models using our dataset. The models were designed using libraries like Tensorflow 2.0, Pandas, Numpy, and OpenCV. The GPU cluster is a one-head node with eight Nvidia RTX 3090s with a GPU memory of 192 GB and a clock boost of 1.70 GHz; the cluster also has a storage of 12 TB for the head node. 

For better convergence during training, we set the learning rate to 0.0001. Since we were dealing with multiclass classification, we employed categorical cross-entropy as our loss function. The loss function denoted with $L_{\mathrm{CCE}}$ is defined as follows:

(7) $$L_{\mathrm{CCE}}=-\frac{1}{N_{m}}\;\;\sum_{i=1}^{N_{m}}\sum_{c=1}^{C}1_{y\;\in {\;C}_{C}\;}{\log(y}_{i}\;\in {\;C}_{C})\;$$

where $N_{m}$ is the number of total images (input), C denotes the number of classes in the multiclass framework, and $y_{i}\;\in {\;C}_{C}$ is the indicator function representing the i^th^ term in the c^th^ category. The models were trained over 50 epochs with a batch size of 16, which allowed for efficient processing of the dataset. To mitigate overfitting, we applied a dropout rate of 0.5. For the activation functions, we utilized softmax for the classification layer and ReLU for the other layers. This software and hardware environment helped to optimally train the DermAI system effectively.

### 3.6. Experimental Protocols

Based on our preliminary analysis, we developed an experimental workflow, which we have mentioned in this section. Initially, we examined our fine-tuned TL models and evaluated their performance. Subsequently, we investigated how we can make ensemble-based models out of our fine-tuned TL models using techniques like softmax voting, weighted average, and stack-based. We then evaluated the impact of the attention mechanism on the models on the skin lesion classification task. We also researched making ensemble-based models using the attention mechanism to see how they affect the classification performance.

#### 3.6.1. Experiment 1: Performance of TL Models Using the HAM10000 Dataset

For this experiment, we utilized seven different types of TL models, namely ResNet101 [43], MobileNet [44], InceptionV3 [45], EfficientNet B3 [46], EfficientNet B7 [46], DenseNet-201 [47], and NASNet Mobile [48]. All these models were pre-trained on the ImageNet dataset. The main focus of the experiment was to showcase the effectiveness of TL models compared to scratch convolutional networks. To effectively predict skin lesions, we used the HAM10000 dataset along with the K10 protocol.

#### 3.6.2. Experiment 2: Comparison of EBDL Models vs. TL Models

The main objective of this study is to examine the effectiveness of an ensemble-based paradigm in skin lesion classification. To achieve this, we designed EBDL models using techniques such as softmax voting, weighted average, and stack-based. We generated *six* EBDL models using these three techniques and compared them with seven TL models. To ensure the reliability of the results, the experiment utilized the K10 cross-validation protocol.

#### 3.6.3. Experiment 3: Comparison of TL Models vs. aeTL Models

In this experiment, our focus was to examine and compare the impact of employing an attention mechanism on TL models for skin lesion classification. To achieve this, we used an attention block and built seven aeTL models, which were then compared with seven non-attention TL models. The study was conducted using constant K10 cross-validation protocols.

#### 3.6.4. Experiment 4: Comparison of aeTL Models vs. aeEBDL Models

In this experiment, our focus was on evaluating the effectiveness of the ensemble-based paradigm in an attention-enabled framework by comparing aeTL and aeEBDL models for skin lesion classification. To achieve this, we built and compared three aeEBDL models along with seven aeTL models. The study was conducted using constant K10 cross-validation protocols.

#### 3.6.5. Experiment 5: Effect of Training Size on the Performance of TL and aeTL Models

To validate the robustness of our TL models in substantially lesser amounts of training data, we analyzed four cross-validation protocols: K2, K4, K5, and the default K10 protocol. Using these protocols, we verified the performance drop of the models with varying training data sizes for each of the seven TL models. By averaging the results across the models, we intend to measure the difference in performance metrics from 90% Training data size to 50% Training data size. 

### 3.7. Performance Metrics

For measuring the performance of the models on various fronts we have used various performance metrics. True positive (TP), true negative (TN), false positive (FP), and false negative (FN) came in handy to estimate the performance. We have used accuracy (η), recall (R), precision $\left(\rho \right)$, and F1-score (F). After calculating the accuracy of the TL, EBDL, aeTL, and aeEBDL models, we calculated their mean accuracies (Δ) in Equations (8–18), respectively. In these equations “m” is the current model, “M” is the number of models, “d” is the current dataset, and “D” is the number of datasets. The probability curve ROC and degree of separability Area-under-the-curve (AUC) have been also calculated for every model. In standard deviation $\left(\sigma \right)$, each value from the population is denoted by x_i_ and μ, the population mean.

## 4. Results

The protocols were employed to evaluate the effectiveness of the system in skin lesion classification with the HAM10000 dataset in a multiclass framework. To analyze the system, a total of 23 AI models were utilized: seven TL models, *six* EBDL models, and seven aeTL models, and *three* aeEBDL models.

(8) $$\eta =\frac{\mathrm{TP}+\mathrm{TN}}{\mathrm{TP}+\mathrm{FP}+\mathrm{FN}+\mathrm{TN}}$$

(9) $$R=\frac{\mathrm{TP}}{\mathrm{TP}+\mathrm{FN}}$$

(10) $$\rho =\frac{\mathrm{TP}}{\mathrm{TP}+\mathrm{FP}}$$

(11) $$F=2\times \frac{p\;*\;R}{p+R}$$

(12) $$\bar{\eta }\left(m,\;K10\right)=\frac{\sum_{d=1\;}^{D}\eta \left(m,\;d,\;K10\right)}{D}$$

(13) $$\bar{\eta }\left(d,\;K10\right)=\frac{\sum_{m=1}^{M}\;\eta \left(m,\;d,\;K10\right)}{M}$$

(14) $${\bar{\eta }}_{\mathrm{sys}}=\frac{\sum_{d=1\;}^{D}\sum_{m=1}^{M}\;\eta \left(m,\;d,\;K10\right)}{M\;\times \;D}$$

(15) $$\bar{\alpha }\left(m,\;K10\right)=\frac{\sum_{d=1\;}^{D}\alpha \left(m,\;d,\;K10\right)}{D}$$

(16) $$\bar{\alpha }\left(d,\;K10\right)=\frac{\sum_{m=1}^{M}\;\alpha \left(m,\;d,\;K10\right)}{M}$$

(17) $${\bar{\alpha }}_{\mathrm{sys}}=\frac{\sum_{d=1\;}^{D}\sum_{m=1}^{M}\;\alpha \left(m,\;d,\;K10\right)}{M\;\times \;D}$$

(18) $$\sigma =\sqrt{\frac{1}{n}\left[\overset{n}{\underset{i=1}{\sum }}{\left(X_{i}-\mu \right)}^{2}\right]}$$

### 4.1. Performance of TL Models Using the HAM10000 Dataset

In this study, we demonstrate the performance of various TL models on the HAM10000 dataset. The experiment involved training and testing seven state-of-the-art baseline TL models of comparable architecture on an augmented dataset. As shown in Table 1, our models achieved a mean accuracy of **95.30%.** Notably, EfficientNet B3 outperformed all other models, achieving an accuracy of **97.01%** with an F1-score of **0.9698**. This exceptional performance can be attributed to the ability of TL models to process skin lesion images and extract essential features for accurate classification.

### 4.2. Comparison of EBDL Models vs. TL Models

In this experiment, we developed six EBDL models using baseline TL models and utilized techniques like softmax voting, weighted average, and stack-based to create these models. The performance of the EBDL models is presented in Table 2, which showcases various performance metrics. The stack-based EBDL6: DenseNet201 + ResNet101 + NASNetMobile model outperformed other models with an accuracy of **99.69%** and an F1-score of **0.9969**. Furthermore, we compared the results of EBDL models with those of solo models, and the EBDL models demonstrated superior performance. Table 3 demonstrates the effectiveness of EBDL techniques in significantly improving the overall classification model, with EBDL techniques resulting in a **4.22%** increase in mean accuracy.

This improvement can be attributed to the fact that EBDL models leverage the strengths of different individual models and compensate for their weaknesses, leading to enhanced accuracy and generalization ability. Moreover, EBDL models can effectively address the limitations of TL models by combining predictions from multiple models, each with its unique strengths and weaknesses, thereby reducing the risk of overfitting and enhancing the robustness of the models.

### 4.3. Comparison of TL Models vs. aeTL Models

In this experiment, we implemented attention mechanisms on seven TL models and evaluated the benefits gained by incorporating attention into these models. According to Table 4 and Table 5, the mean accuracy of the aeTL models is **98.31%**, showing an average increase of **3.01%** compared to the TL models. Among the aeTL models, aeEfficientNet B3 achieved the highest individual accuracy of **98.96%** and an F1-score of **0.9896**. This attention block proved to be most beneficial for the ResNet101 model, which experienced a significant increase of **4.10%** in accuracy after incorporating attention. These results further support the hypothesis that attention blocks are a powerful paradigm in classification tasks.

This successful application of attention mechanisms on TL models demonstrates their ability to improve the models’ performance, resulting in higher accuracy and better adaptation to the dataset. Due to their capability to focus on important features, they allow the models to better distinguish and classify complex patterns within the data.

### 4.4. Comparison of aeTL Models vs. aeEBDL Models

In this experiment, we developed three aeEBDL models that incorporated solo aeTL models. The results are presented in Table 6, with the best aeEBDL model achieving an accuracy of **99.73%** and an F1-score of **0.9973.** Upon comparing the performance with the seven aeTL models, as shown in Table 7, we observed that aeEBDL models outperform the aeTL models with a mean accuracy increase of **1.2%**. This validates the performance gain in the ensemble-based paradigm, even in an attention-ensembled framework, where aeEBDL leverages the strengths of different individual models and compensates for their weaknesses, leading to enhanced accuracy and generalization ability.

### 4.5. Effect of Training Size on the Performance of TL and aeTL Models

In this experimental research, we examined how different sizes of training data affect the performance of TL and aeTL models. We assessed the performance metrics using various cross-validation protocols: K10 (default), K5, K4, and K2. The outcomes, presented in Table 8 and Table 9, revealed a gradual decrease in performance metrics across these protocols. Both the best TL model (EfficientNet B3) and the best aeTL model (aeEfficientNet B3) were tested. The computed average mean accuracy demonstrated a decline in mean accuracy from **97.01%** in K10 to **91.39%** in K2, signifying a **5.62%** reduction for EfficientNet B3. Likewise, for aeEfficientNet B3, the accuracy declined from **98.96%** in K10 to **92.68%** in K2, a **6.28%** decrease.

Despite the reduced training data in the K2 (50:50) validation protocol, our AI models exhibited reliable performance metrics. This finding highlights the effectiveness of our approach and how our models demonstrated robust performance even when faced with limited training data, proving their ability to maintain consistent performance under such conditions.

## 5. Performance Evaluation

During the performance evaluation, we utilized ROC curves to visualize the performance of the AI models. Furthermore, we measured the stability of the system by conducting two statistical tests: Wilcoxon signed-rank test and Cohen’s Kappa coefficient on all the models. Also, we conducted a reliability test on all the AI models using misclassification rates of the images.

### 5.1. Receiver Operating Characteristics

**Figure 15:**
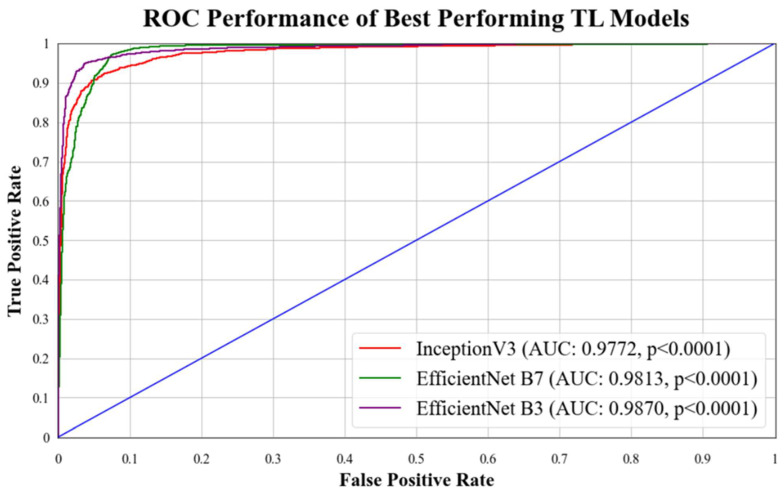
ROC chart of best-performing TL models. Blue line: It denotes random classifier at an AUC of 0.5.

**Figure 16:**
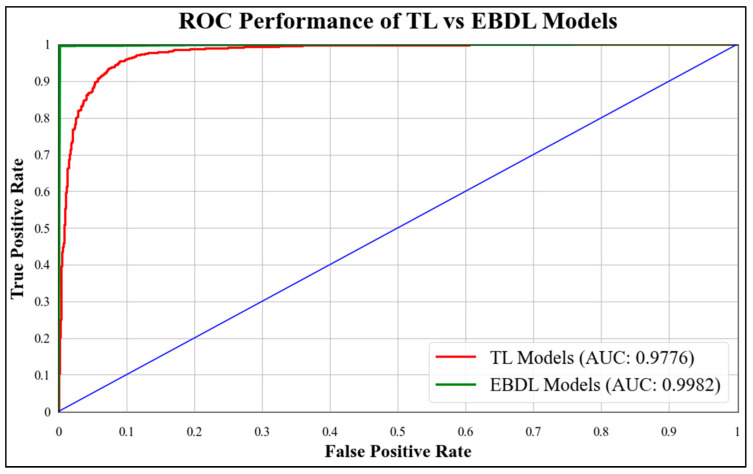
ROC chart of the mean performance of TL vs. EBDL models.

**Figure 17:**
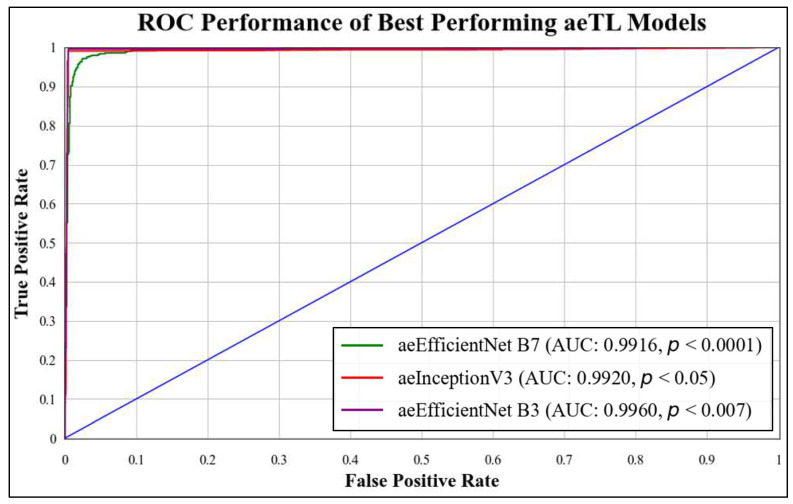
ROC chart of best-performing aeTL models.

ROC curves are commonly used to evaluate the performance of models across their entire operating range. In this study, we have plotted multiple ROC curves to assess the performance of different models. Figure 15 displays the ROC curve for the best-performing TL model, EfficientNet B3, which achieved an AUC of **0.9870**. Figure 16 compares the ROC curves of the mean AUC of TL models (**0.9776**) with EBDL models (**0.9982**). Figure 17 visualizes the ROC curves of the best-performing aeTL models, with aeEfficientNet.

**Figure 18:**
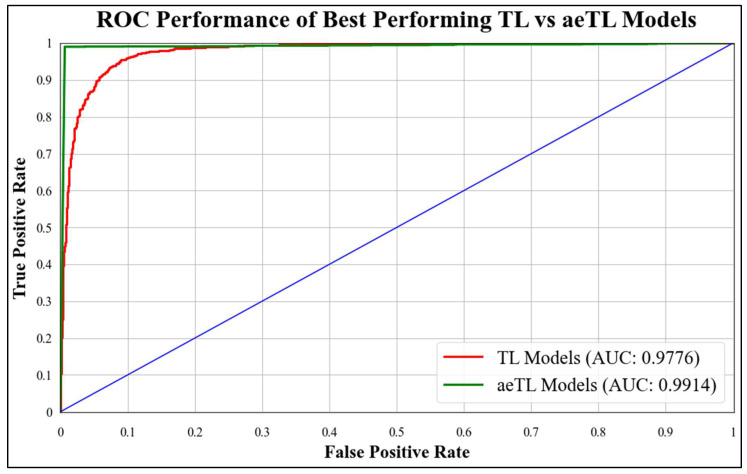
ROC chart of best-performing TL vs. aeTL models.

**Figure 19:**
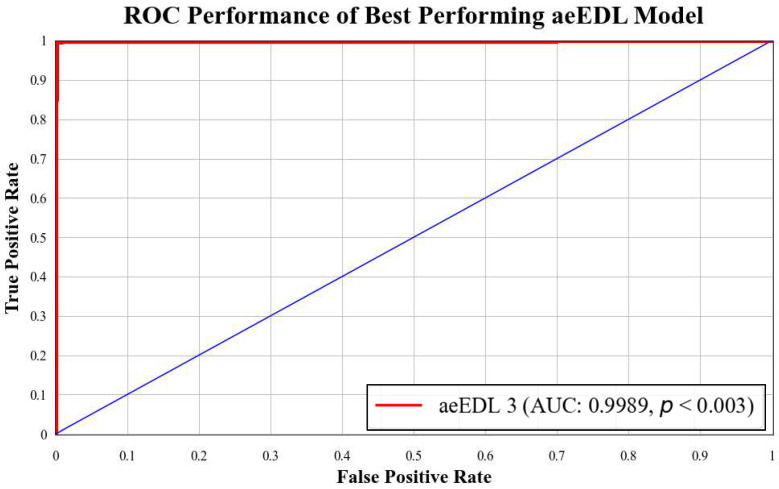
ROC chart of best-performing aeEBDL model.

**Figure 20:**
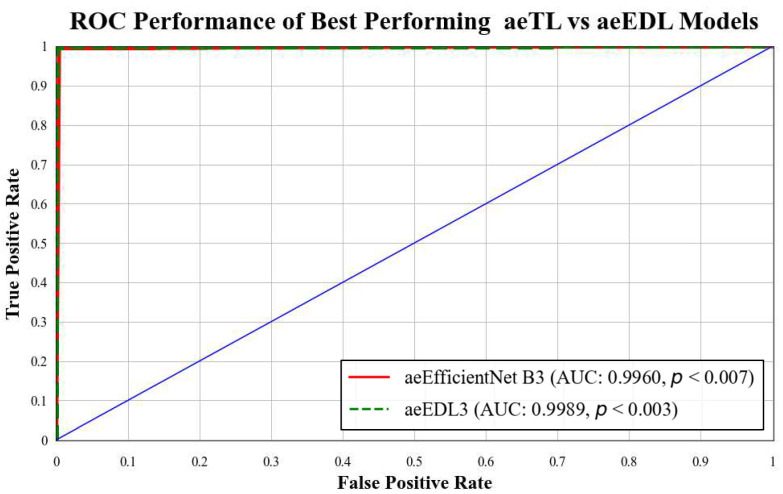
ROC chart of the mean performance of aeTL vs. aeEBDL models.

B3 has an AUC of **0.9960**. Figure 18 presents a comparison of the mean AUC of TL models (**0.9776**) with aetTL models (**0.9914**). Figure 19 portrays the AUC of the overall best-performing model, aeEBDL 3, which achieved an AUC of **0.9989**. Lastly, Figure 20 compares the ROC curves of the best-performing aeTL model (aeEfficientNet B3) with the best-performing aeEBDL model (aeEBDL 3).

Additionally, to establish the statistical significance of the results for all classes in each dataset, *p*-values were calculated. The obtained *p*-values were less than 0.01, indicating a high level of confidence in the observed text classification results. Overall, the use of ROC curves and *p*-values strengthens the evaluation of the models and provides valuable insights into their performance.

### 5.2. Stability Validation Using Statistical Tests

The stability of the system was validated by conducting two statistical tests on all the models. The tests performed were the Wilcoxon signed-rank test and Cohen’s Kappa coefficient, which provide insights into the system’s stability. The Wilcoxon signed-rank test is a non-parametric test used to confirm statistical hypotheses. It compares the locations of two separate populations in data using two matched samples to measure the paired difference. Cohen’s Kappa coefficient is a statistical measure used to assess the reliability of two raters for categorical features. The results of these tests are presented in Table 10. The significance of the predicted labels is identified with *p*-values < 0.001. Furthermore, all the Kappa values ranged between 0.9 and 1.0, indicating an almost perfect agreement among the classifiers.

### 5.3. Reliability Analysis through Misclassification Results

For the reliability assessment of the system, we conducted predictions on the entire dataset using all available models. Subsequently, we computed the predictions made using each model for every image. By comparing these predicted labels with the true labels, we calculated the number of misclassified labels, visualized through Equations (19)–(21). This analysis allowed us to infer the probability of each image being misclassified and identify the types of lesion images that were most challenging to classify.

**Figure 21:**
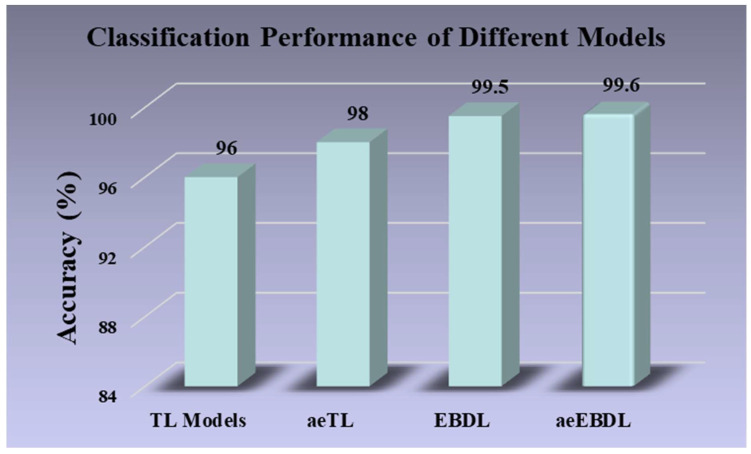
Classification performance of different models.

As shown in Figure 21, the misclassification rate for all the augmented images was approximately **2%** across twenty-three models. However, TL models exhibited a higher misclassification rate of **4%** for images. Also, incorporating attention with TL models reduced the misclassification of images to **2%**. Notably, EBDL models and aeEBDL models demonstrated superior performance by misclassifying only **0.5%** and **0.4%** of images, respectively.

(19) $$\bar{R}=\left(1-{\bar{M}}_{class}\right)\times 100$$

(20) $${\bar{M}}_{class}=\;\frac{\sum_{i=1}^{I}P_{i}}{I}$$

(21) $$P_{i}=\frac{{\#\;\mathrm{of}\;\mathrm{misclassification}\;\mathrm{for}\;i}^{\mathrm{th}}\;\mathrm{image}\;\mathrm{over}\;M\;\mathrm{models}}{M}$$

where, $\bar{R}$ is the reliability index, ${\bar{M}}_{class}$ is the misclassification value, and I is the total number of images.

## 6. Discussion

The proposed system has been trained on the HAM1000 dataset and incorporates superior quality control techniques and class balancing using augmentation. We implemented seven TL models with and without attention, as well as six EBDL and three aeEBDL models on the dataset. Based on our comprehensive analysis, we summarize the primary and secondary findings. Through extensive experimentation and model analysis, we have proven our hypothesis. Additionally, we have benchmarked our proposed techniques (aeTL and aeEBDL) against existing studies in the field of skin cancer classification. Furthermore, the incorporation of attention mechanisms in our models proved to be advantageous. The attention paradigms enhanced the models’ ability to capture important features, resulting in a more accurate and robust system.

### 6.1. Principal Findings

Extensive experimentation was conducted on various models, leading to valuable insights into their performances and effectiveness. (i) Incorporating ensemble-based architectures (max voting, weighted ensemble, and stack) increased the mean accuracy of EBDLs by **4.22%** compared to their component TL models. (ii) aeTL models exhibited better performance compared to baseline TL models increasing the mean accuracy by **3.01%**. (iii) aeEBDL models outperformed aeTL models in terms of accuracy, efficiency, and robustness, leading to a mean accuracy increase of **1.29%**. (iv) The proposed models validated the hypotheses and demonstrated their behavior on data augmentation and class balancing. (v) Fine-tuned TL models initially outperformed existing approaches, with the fine-tuned EfficientNet B3 achieving the highest accuracy of **97.01%** on the augmented dataset, while ResNet-101 achieved the lowest accuracy of **94.07%** on the stack. (vi) Incorporating attention in the architecture further improved classification performance, with aeResNet-101 showing the highest accuracy increase of **4.10%** when adapted to attention. (vii) The proposed models were validated through statistical tests and analyses of the effect of the training sample size on training accuracy. (viii) An elaborate and novel misclassification paradigm was utilized to inspect reliability of the proposed models. (ix) The proposed system achieved superior performance over the benchmark in the domain, by a significant margin.

The methodologies presented in this research paper are advanced and effective techniques for the classification of skin lesions.

### 6.2. Benchmarking

We studied several papers and sorted some of the latest papers for benchmarking. The crux of our research was positioned using an attention-enabled paradigm in skin lesion classification. Seven state-of-the-art models, including ResNet-101, MobileNet, InceptionV3, EfficientNet B3, EfficientNetB7, DenseNet-201, and NASNet Mobile have been used on the HAM10000 dataset followed by the formation of EBDLs, aeTLs, and aeEBDLs. We evaluated the models based on their accuracy, precision, recall, F1-score, *p*-value, and AUC and compared the results to the previous benchmark studies. In our experimental results, our proposed aeEfficientNet B3 (in solo TL models) outperformed all other models with an accuracy of **98.96%**, with a precision, recall, and F1-score of **99%**. Our proposed aeEBDL-3, which used ResNet-101, MobileNet, InceptionV3, EfficientNet B3, EfficientNetB7, and DenseNet-201 outperformed all other models with an accuracy of **99.73%**, with a precision, recall, and F1-score of **100%**. The second-best model was an EBDL model by Kauser et al. [49] based on ResNet, InceptionV3, DenseNet, InceptionResNetV2, and VGG 19 and achieved an accuracy of **98%**, with precision, recall, and F1-score of **99%**. We have also compared our proposed models with other existing models by Hoang et al. [50], Chaturvedi et al. [51], Ali et al. [39], Rahi et al. [52], Lim et al. [53], Moldovan et al. [54], Iqbal et al. [55], and Charan et al. [56], who achieved accuracies of **84.80%**, **93.2%**, **90.60%**, **90%**, **83.23%**, **80%**, **88.75%**, and **88.6%**, **respectively**.

Table 11 focused on eight studies that focused on building classifiers for skin lesion classification. Moldovan et al. [54] presented a dual-step classification approach based on TL. They used a DenseNet121 pre-trained model to predict the half number of classes in the first step and the other remaining classes in the second step for the overall classification. Lim et al. [53] used a fine-tuned MobileNet TL model and compared their models with their performance on augmented and non-augmented data. Rahi et al. [52] based the idea on the usage of CNN and TL models for classification and conducted a comparison of the performance of models. They used ResNet50, DenseNet121, and VGG11 as pre-trained models and made their CNN from scratch, then evaluated their results. Chaturvedi et al. [51] proposed a fine-tuned ResNetXt101 for the classification of skin lesions and conducted a comparative study of five state-of-the-art TL models and constructed four EBDL models out of them. Iqbal et al. [55] demonstrate their deep CNN models that outperforms the state-of-the-art models for the classification on ISIC-19 data. Ali et al. [39] used normalization and data augmentation paradigms to train their proposed DCNN model that outperforms TL models such as AlexNet, VGG16, and ResNets. Kauser et al. [49] proposed EBDL models using TL models like InceptionResNetV2 as base models and used majority voting and weighted majority voting techniques for their classifiers. Charan et al. [56] proposed a two-path neural network, with one path taking input as original images and the second with multiple original images and their segmentation mask for the classification. Hoang et al. [47] proposed a entropy-based weighting and first-order cumulative combined segmentation technique to segment the HAM10000 dataset and further used these images for the channel shuffling function to train their proposed wide ShuffleNet for classification.

Our results showcase the effectiveness of our best-in-class models, which outperform these existing models and demonstrate the effectiveness of aeTLs and aeEBDL. Our study also highlights the effectiveness of data augmentation methodology in medical imaging for increasing the training sample size, effectiveness, and robustness of a model.

### 6.3. Special Note on the Use of Attention in Skin Lesion Classification

Attention mechanisms play a vital role in enhancing skin lesion detection by enabling models to selectively focus on important features of the input image for classification, rather than treating every image part equally. This utilization of attention proves particularly advantageous in skin lesion classification as it allows the models to concentrate selectively on lesion areas, rather than the surrounding normal skin, facilitating the identification of the required features for accurate classification.

Moreover, attention mechanisms assist the models in improving feature extraction by prioritizing relevant features over irrelevant ones. This emphasis on relevant features enhances the model’s performance and leads to a more accurate classification of skin lesions. By employing attention mechanisms, the models can effectively discern the distinguishing characteristics of skin lesions, resulting in a more robust and precise classification system.

### 6.4. Special Note on the Use of Segmentation of Lesion

While using a segmentation-based approach can be reliable and effective in identifying lesions in medical images [57,58,59,60,61], we chose not to implement it for several reasons. Our main objective was multiclass classification, and we discovered that we were able to achieve accurate results without the need for segmentation. The lesion sizes in our images were already relatively large, and the region of interest (ROI) around the lesion was closely positioned and centered, resulting in smaller background regions. This allowed our classification models to focus on relevant features without the requirement of external lesion segmentation.

Additionally, when we experimented with incorporating segmentation, we observed only a minor 1–2% overall improvement in classification performance, which was not significant enough to justify the added complexity and computational resources it entailed. By solely focusing on the classification task, we streamlined and pipelined our approach to achieve accurate results more efficiently.

Our classification models effectively learned to distinguish between different lesion classes, and just omitting the segmentation phase did not hinder their performance. This decision enabled us to allocate more resources to optimizing the classification models and exploring the attention-enabled and ensemble-based learning paradigm, ultimately leading to successful lesion detection without relying on segmentation. While segmentation was not the primary objective, segmentation methods based on level sets can be possible solutions for lesion segmentation [62,63,64,65]. Classifications that combine deep learning-based features with machine learning for classifiers [66] have also proved effective.

### 6.5. Special Strengths, Weakness, and Extension

The research focused on the application of TL, EBDL, aeTL, and aeEBDL methods in skin lesion classification. The study showed significant improvement in the classification process, making the proposed methods a benchmark in the fields for skin lesion classification. The proposed models outperform existing studies. The use of attention in models gave an upper hand in classification, compared to existing models, and the EBDL technique leveraged the trained models’ capabilities for increasing the prediction accuracy. Additionally, cross-validation and statistical tests prove the system’s robustness and domain adaptability.

Due to the limited availability of high-quality dermatoscopic images, the existing study focused mainly on training classifiers on specific datasets. The HAM10000, although extensive, still has limited variation in representing the full spectrum of skin cancer or pigmented skin lesions. Also, the imbalanced class distribution potentially will overlook other types of skin cancer. The system requires an extra step of augmentation, which may introduce synthetic samples into the training data, inheriting uncertainties and limitations.

The exploration of generalizing the models to adapt more to the domain and regularization techniques such as weight decay can help in reducing the overfitting of AI models to datasets and their ability to generalize more to unseen and clinical data. [67,68,69] There have been studies in different domains, such as immunology [70,71], cardiovascular risk assessment [72], psoriasis diagnosis [73], and thyroid Doppler [74], where cloud-based end-to-end systems are used for detection and classification. We therefore intend to deploy our models on a similar paradigm to create an automated, scalable, and accessible skin cancer classification that will allow easy integration with other applications, remote access, and effective use of computational resources [71,75]. The proposed cloud-based system follows a layered architecture, where the presentation layer operates locally on the users’ devices, while the business and persistence layers are hosted on the cloud. This ensures a user-friendly system providing a real-time classification system on the device itself [72]. Moreover, the design systems can be pruned to reduce the size of the trained models, leading to less consumption of computational resources and memory requirements [76,77]. Artificial Intelligent systems are prone to bias, which can emerge due to imbalanced data distributions, demographic disparities, or variations in data collection protocol. Techniques involving bias mitigation and ranking them according to their bias can be introduced into the system [78,79,80]. Lastly, adopting transformer-based architectures [81] for their excellence in capturing long-range dependencies and feature information can be beneficial for the detection of pigmented skin lesions. Vision transformers [82] will be a healthy approach in handling large-image resolutions, making more interpretable attention maps or enhancing the scalability and parallel processing powers.

Another area that can improve the classification is the adoption of automated skin lesion segmentation using advanced segmentation methods such as level sets [65,83] or stochastic medical image analysis methods [64].

## 7. Conclusions

Our study demonstrates a novel paradigm for skin lesion classification based on image input. The system employs seven TL models, six EBDL models derived from the solo TL models, and seven aeTL models, along with three aeEBDL models. We designed a comprehensive experimental structure to assess model performance and validate our hypothesis. The system’s generalization and reliability were evaluated through performance assessments using ROC curves, statistical tests, and reliability analyses through misclassification. Furthermore, we developed a benchmarking strategy concerning related strategies for skin lesion classification. The proposed system demonstrated high reliability and stability.

## Figures and Tables

**Figure 4:**
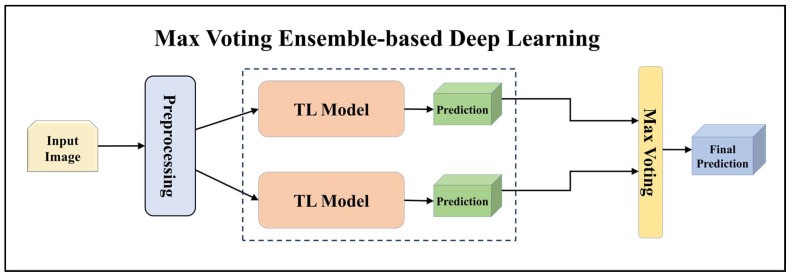
Architecture of max voting ensemble-based deep learning model.

**Figure 5:**
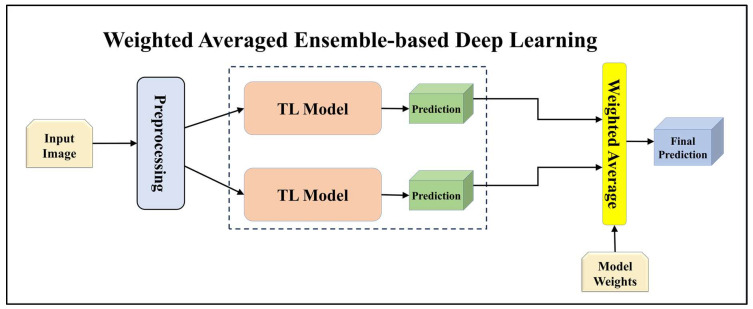
Architecture of weighted averaged ensemble-based transfer learning model.

**Figure 6:**
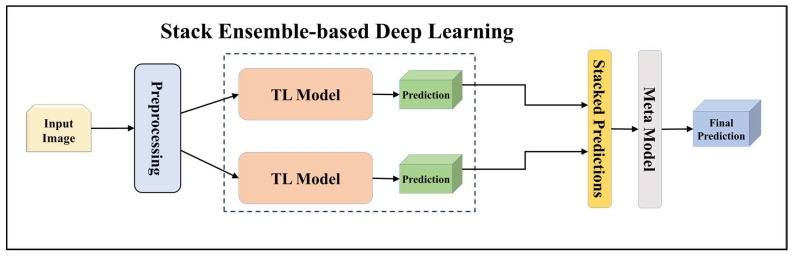
Architecture of stack ensemble-based deep learning model.

**Figure 7:**
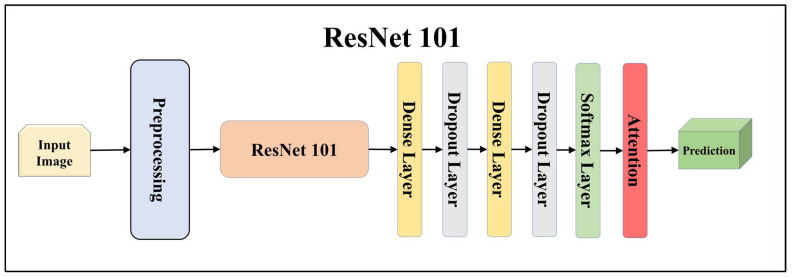
Architecture of attention-enabled ResNet 101 model.

**Figure 8:**
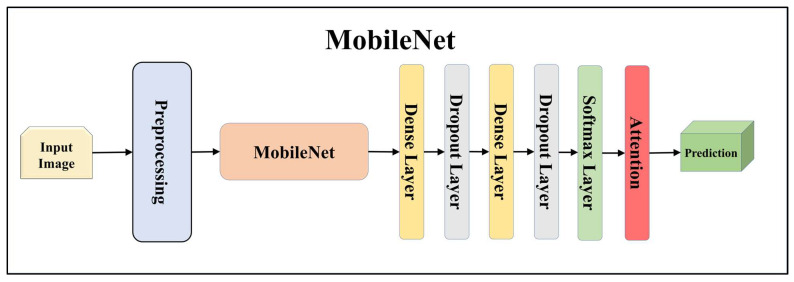
Architecture of attention-enabled MobileNet model.

**Figure 9:**
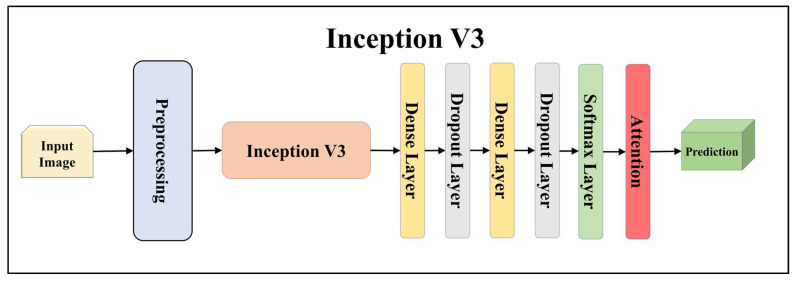
Architecture of attention-enabled Inception V3 model.

**Figure 10:**
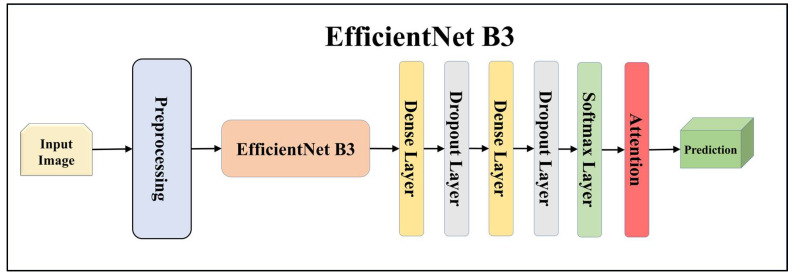
Architecture of attention-enabled EfficientNet B3 model.

**Figure 11:**
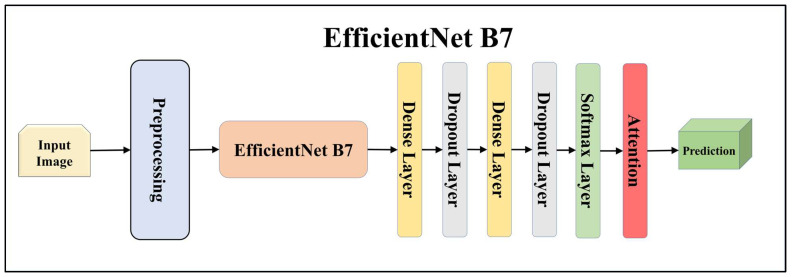
Architecture of attention-enabled EfficientNet B7 model.

**Figure 12:**
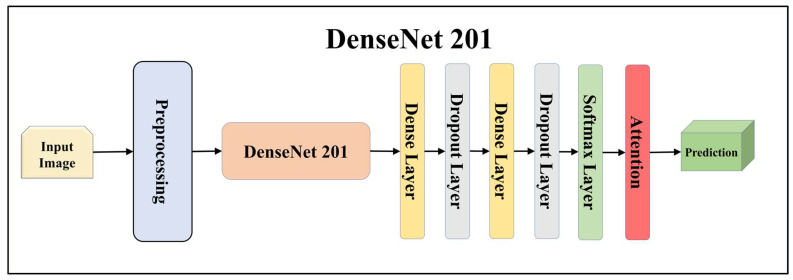
Architecture of attention-enabled DenseNet 201 model.

**Figure 13:**
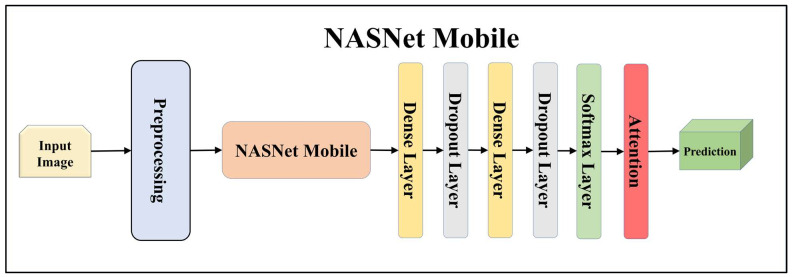
Architecture of attention-enabled NASNet Mobile model.

**Figure 14:**
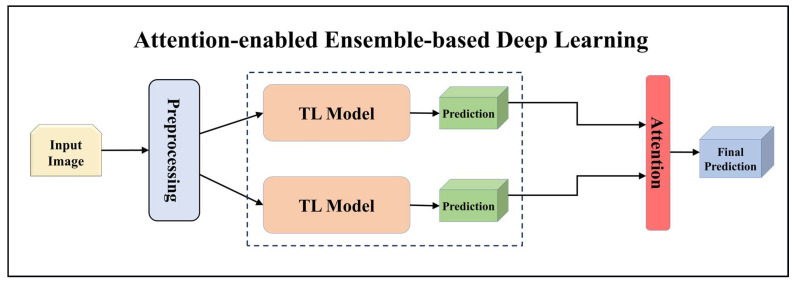
Architecture of attention-enabled ensemble-based deep learning model.

**Table 1:** Performance metrics of seven TL models.

Performance Metrics of seven TL Models							
TL	TL Model	Accuracy (%)	AUC [0–1]	Precision [0–1]	Recall [0–1]	F1-Score [0–1]	S.D. (%)
TL 1	ResNet 101	94.10%	0.9697	0.9428	0.9407	0.9406	1.51
TL 2	MobileNet	94.73%	0.9748	0.9501	0.9484	0.9482	1.5
TL 3	NASNet Mobile	94.93%	0.9755	0.9508	0.9488	0.9485	1.48
TL 4	DenseNet 201	95.18%	0.9780	0.9535	0.9523	0.9518	1.51
TL 5	Inception V3	95.41%	0.9772	0.9550	0.9542	0.9539	1.49
TL 6	Efficient Net B7	95.76%	0.9813	0.9589	0.9565	0.9558	1.49
**TL 7**	**Efficient Net B3**	**97.01%**	**0.9870**	**0.9701**	**0.9700**	**0.9698**	**1.51**
**Mean accuracy of all models = 95.30%**							

**Table 2:** Performance metrics of six EBDL models.

Performance Metrics of *six* EBDL Models								
EBDL	Ensemble Technique	Base Models	Accuracy (%)	AUC [0–1]	Precision [0–1]	Recall [0–1]	F1-Score [0–1]	S.D. (%)
EBDL 1	Weighted Average	MobileNet + EfficientNet B3 + InceptionV3	99.11%	0.9984	0.9960	0.9960	0.9960	1.51
EBDL 2	Max Voting	NASNet Mobile + ResNet101 + EfficientNet B7	99.34%	0.9972	0.9934	0.9934	0.9933	1.50
EBDL 3	Max Voting	ResNet101 + MobileNet + InceptionV3	99.58%	0.9981	0.9958	0.9958	0.9958	1.51
EBDL 4	Weighted Average	MobileNet + EfficientNet B7 + ResNet101 + DenseNet201	99.68%	0.9987	0.9968	0.9968	0.9967	1.51
EBDL 5	Stack	ResNet101 + MobileNet + InceptionV3	99.69%	0.9986	0.9969	0.9969	0.9969	1.51
**EBDL 6**	**Stack**	**DenseNet201 + ResNet101 + NASNet Mobile**	**99.69%**	**0.9987**	**0.9969**	**0.9969**	**0.9969**	**1.51**
**Mean accuracy of all models = 99.52%**								

**Table 3:** Comparison of EBDL models vs. TL models.

Comparison of EBDL Models vs. TL Models					
Model Type	Mean Accuracy (%)	Mean AUC [0–1]	Mean Precision [0–1]	Mean Recall [0–1]	Mean F1-Score [0–1]
TL Models	95.30%	0.9776	0.9544	0.9529	0.9526
EBDL Models	99.52%	0.9982	0.9959	0.9959	0.9959
**Mean Increase (%)**	**4.22%**	**2.06%**	**4.15%**	**4.30%**	**4.33%**

**Table 4:** Performance metrics of seven aeTL models.

Performance Metrics of Seven aeTL Models							
aeTL	TL Model	Accuracy (%)	AUC [0–1]	Precision [0–1]	Recall [0–1]	F1-Score [0–1]	S.D. (%)
aeTL 1	ResNet 101	98.17%	0.9908	0.9823	0.9817	0.9818	1.52
aeTL 2	MobileNet	98.21%	0.9919	0.9824	0.9821	0.9821	1.51
aeTL 3	NASNet Mobile	97.45%	0.9871	0.9750	0.9745	0.9745	1.49
aeTL 4	DenseNet 201	97.91%	0.9906	0.9792	0.9791	0.9790	1.51
aeTL 5	Inception V3	98.56%	0.9920	0.9858	0.9856	0.9856	1.5
eTL 6	Efficient Net B7	98.96%	0.9916	0.9816	0.9804	0.9805	1.52
**aeTL 7**	**Efficient Net B3**	**98.96%**	**0.996**	**0.9897**	**0.9896**	**0.9896**	**1.52**
**Mean accuracy of all models = 98.31%**							

**Table 5:** Comparison of TL models vs. aeTL models.

Comparison of TL Models vs. aeTL Models					
Model Type	Mean Accuracy (%)	Mean AUC [0–1]	Mean Precision [0–1]	Mean Recall [0–1]	Mean F1-Score [0–1]
TL Models	95.30%	0.9776	0.9544	0.9529	0.9526
aeTL Models	98.31%	0.9914	0.9822	0.9818	0.9818
**Mean Increase (%)**	**3.01%**	**1.38%**	**2.78%**	**2.89%**	**2.92%**

**Table 6:** Performance metrics of three aEBDL models.

Performance Metrics of aeEBDL Models								
EBDL	Ensemble Technique	Base Models	Accuracy (%)	AUC [0–1]	Precision [0–1]	Recall [0–1]	F1-Score [0–1]	S.D. (%)
aeEBDL 1	Attention-enabled	ResNet101+ MobileNet + InceptionV3	99.58%	0.9981	0.9958	0.9958	0.9958	1.51
aeEBDL 2	Attention-enabled	MobileNet + InceptionV3 + NASNet Mobile	99.49%	0.9977	0.9949	0.9949	0.9949	1.50
aeEBDL 3	Attention-enabled	ResNet101 + MobileNet + InceptionV3 + EfficientNet B3 + EfficientNet B7 + DenseNet201	99.73%	0.9989	0.9973	0.9973	0.9973	1.51
**Mean accuracy of all models = 99.60%**								

**Table 7:** Comparison of aeTL models vs. aeEBDL models.

Comparison of aeTL Models vs. aeEBDL Models					
Model Type	Mean Accuracy (%)	Mean AUC [0–1]	Mean Precision [0–1]	Mean Recall [0–1]	Mean F1-Score [0–1]
aeTL Models	98.31%	0.9914	0.9822	0.9818	0.9818
aeEBDL Models	99.60%	0.9982	0.996	0.996	0.996
**Mean Increase**	**1.29%**	**0.68%**	**1.38%**	**1.42%**	**1.42%**

**Table 8:** Effect of training data on performance for EfficientNet B3 model.

Effect of Training Data on Performance for EfficientNet B3 Model							
Model Type	K2	K4	K5	K10	D1 (%)	D2 (%)	D3 (%)
Accuracy (%)	91.39%	94.85%	95.69%	97.01%	1.32%	2.16%	5.62%
AUC [0–1]	0.935	0.9592	0.9744	0.987	1.26%	2.78%	5.20%
Precision [0–1]	0.9002	0.9259	0.9611	0.9701	0.90%	4.42%	6.99%
Recall [0–1]	0.8978	0.9258	0.9614	0.97	0.86%	4.42%	7.22%
F1-Score [0–1]	0.899	0.9258	0.9612	0.9698	0.86%	4.40%	7.08%

**D1**: Absolute difference in performance metrics between K10 and K5 cross-validation protocols. **D1 = K10** − **K5**. **D2**: Absolute difference in performance metrics between K10 and K4 cross-validation protocols. **D2 = K10** − **K4**. **D3**: Absolute difference in performance metrics between K10 and K2 cross-validation protocols. **D3 = K10** − **K2.**

**Table 9:** Effect of training data on performance for aeEfficientNet B3 model.

Effect of Training Data on Performance for aeEfficientNet B3 Model							
Model Type	K2	K4	K5	K10	D1 (%)	D2 (%)	D3 (%)
Accuracy (%)	92.68%	94.83%	98.15%	98.96%	0.81%	4.13%	6.28%
AUC [0–1]	0.9416	0.9561	0.9824	0.996	1.36%	3.99%	5.44%
Precision [0–1]	0.9237	0.9629	0.9805	0.9897	0.92%	2.68%	6.60%
Recall [0–1]	0.9235	0.964	0.9801	0.9896	0.95%	2.56%	6.61%
F1-Score [0–1]	0.9236	0.9634	0.9803	0.9896	0.93%	2.62%	6.60%

**D1**: Absolute difference in performance metrics between K10 and K5 cross-validation protocols. **D1** = **K10** − **K5. D2**: Absolute difference in performance metrics between K10 and K4 cross-validation protocols. **D2 = K10** − **K4**. **D3**: Absolute difference in performance metrics between K10 and K2 cross-validation protocols. **D3 = K10** − **K2.**

**Table 10:** Results of conducted statistical tests on 23 AI models. TL: transfer learning; EBDL: ensemble deep learning; aeTL: attention-based transfer learning; and aeEBDL: attention-based ensemble deep learning.

Model Type	Model Name	Wilcoxon *p*-Test	Cohen’s Kappa
**TL** **Models**	ResNet 101	*p* < 0.001	0.9251
	MobileNet	*p* < 0.001	0.9348
	NASNet Mobile	*p* < 0.001	0.9352
	DenseNet 201	*p* < 0.001	0.9398
	Inception V3	*p* < 0.001	0.9422
	Efficient Net B7	*p* < 0.001	0.9451
	Efficient Net B3	*p* < 0.001	0.9622
**EBDL** **Models**	EBDL 1	*p* < 0.004	0.995
	EBDL 2	*p* < 0.001	0.9917
	EBDL 3	*p* < 0.001	0.9948
	EBDL 4	*p* < 0.002	0.996
	EBDL 5	*p* < 0.06	0.9962
	EBDL 6	*p* < 0.007	0.9962
**aeTL** **Models**	aeResNet 101	*p* < 0.001	0.9769
	aeMobileNet	*p* < 0.001	0.9774
	aeNASNet Mobile	*p* < 0.001	0.9679
	aeDenseNet 201	*p* < 0.001	0.9736
	aeInception V3	*p* < 0.05	0.9819
	aeEfficient Net B7	*p* < 0.001	0.9753
	aeEfficient Net B3	*p* < 0.007	0.9869
**aeEBDL** **Models**	aeEBDL 1	*p* < *0.001*	0.9948
	aeEBDL 2	*p* < 0.001	0.9936
	aeEBDL 3	*p* < *0.003*	0.9967

**Table 11:** Benchmarking of studies that were implemented for skin lesion classification.

Year	Author	AI Models	Approach	Methodology	Dataset	Accuracy (%)	Precision (%)	Recall (%)	F1 (%)	AUC [0,1]	Scientific Val.	Clinical Val.
2019	Moldovan et al. [54]	DenseNet-121	TL	Dual-step CNN Based on DenseNet121	HAM 10000	80	**🗴**	**🗴**	**🗴**	**🗴**	**🗴**	**🗴**
2019	Lim et al. [53]	MobileNet	TL	Augmented Data on Fine-tuned MobileNet	HAM 10000	83.23	**🗴**	**🗴**	82	**🗴**	**🗴**	**🗴**
2019	Rahi et al. [52]	ResNet-50	TL	Fine-tuned TL Models and a CNN from Scratch	HAM 10000	90	91	89	89	**🗴**	**🗴**	**🗴**
2020	Chaturvedi et al. [51]	IRv2	TL	Fine-tuned ResNetXt101	HAM 10000	93.2	87	88	**🗴**	**🗴**	**🗴**	**🗴**
2021	Iqbal et al. [55]	DCNN	CNN	Deep CNN Models	ISIC-19	88.75	90.66	**🗴**	89.75	0.95	**🗴**	**🗴**
2021	Ali et al. [39]	DCNN	CNN	Normalization and Data Augmentation with DCNN	HAM 10000	90.16	94.63	93.91	94.27	**🗴**	**🗴**	**🗴**
2021	Kauser et al. [49]	Ensemble-based Model	EBDL	Fine-tuned InceptionResNetV2 with Ensemble	ISIC-19	98	99	99	99	**🗴**	**🗴**	**🗴**
2022	Charan et al. [56]	Two-path CNN	CNN	Dual-input CNN	HAM 10000	88.6	**🗴**	**🗴**	**🗴**	**🗴**	**🗴**	**🗴**
2022	Hoang et al. [50]	ShuffleNet	TL	Segmentation-based ShuffleNet	HAM 10000	84.8	75.15	**🗴**	72.61	**🗴**	**🗴**	**🗴**
2023	Proposed Study	EfficientNet B3 + Attention	TL	Fine-tuned EfficientNetB3 with Attention	HAM 10000	98.96	99	99	99	0.996	*p* < 0.007	**🗴**
2023	Proposed Study	Ensemble-based Model	EBDL	Ensemble Model of TL models with Attention	HAM 10000	99.73	100	100	100	0.9989	*p* < 0.003	**🗴**

**🗴**: It means that the particular column was not done/ conducted by the studies.

## Data Availability

Due to propriety nature, supporting data cannot be made available openly.

## Acronym Table

**SN**	**Abb ***	**Definition**	**SN**	**Abb ***	**Definition**
1	ACT	Active contour technique	12	GLCM	Gray Level Co-occurrence Matrix
2	aeEBDL	Attention-enabled EBDL	13	ISU	Idaho State University
3	aeTL	Attention-enabled transfer learning	14	KNN	k-Nearest Neighbors
4	AUC	Area-under-the-curve	15	ML	Machine Learning
5	CNN	Convolutional Neural Network	16	NASNet	Neural Architecture Search Network
6	DCNN	Deep CNN	17	ROI	Region of interest
7	DL	Deep Learning	18	ROC	Receiver operating characteristics
8	DT	Decision Trees	19	SVM	Support Vector Machine
9	EBDL	Ensemble-based Deep Learning	20	TL	Transfer Learning
10	FP	False Positive	21	TP	True Positive
11	FN	False Negative	22	TN	True Negative
Abb * = abbreviation.

## Symbol Table

**SN**	**Symbols**	**Explanation**
1	**akiec**	Actinic keratoses and intraepithelial carcinoma
2	**bcc**	Basal cell carcinoma
3	**bkl**	Benign keratosis-like lesions
4	***df***	Dermatofibroma
5	**mel**	Melanoma
6	**nv**	Melanocytic nevi
7	**vasc**	Vascular lesions
8	η	Accuracy
9	R	Recall
10	P	Precision
11	F	F1-Score
12	M	Total models that were used in the system
13	D	Total datasets that were used in the system
14	m	Current model that is being studied
15	d	Current dataset that is being studied
16	${\;p}_{m}$	Denotes the m^th^ model’s prediction score containing the prediction of each class
17	$o_{m}$	Denotes the m^th^ model’s attention output score containing the prediction of each class
18	$w_{m}$	Denotes the m^th^ model’s attention weight
19	C	Denotes the number of classes in the multiclass framework
20	$p_{aeEBDL}$	Is the final attention-enabled and ensemble-based model’s output
21	$\bar{\eta }\left(m,\;K10\right)$	Accuracy of model “m” over all D datasets over the K10 protocol
22	$\bar{\eta }\left(d,\;K10\right)$	Accuracy achieved over the dataset “d” over all M Models over the K10 protocol
23	${\bar{\eta }}_{\mathrm{sys}}$	Overall system accuracy over M models and D datasets
24	$\bar{\alpha }\left(m,\;K10\right)$	AUC of model m summarized over all D datasets
25	$\bar{\alpha }\left(d,\;K10\right)$	AUC achieved over dataset d over all M Models
26	${\bar{\alpha }}_{\mathrm{sys}}$	Overall system AUC over M models and D datasets
27	σ	Standard Deviation of the system
28	$\bar{R}$	Mean Reliability Index of the system
29	${\bar{M}}_{class}$	Mean Misclassification value
30	$p_{i}$	Mean of Misclassification of i^th^ image over all AI models
31	I	Total number of images for misclassification probability

## References

[1] Clark W.H., Elder D.E., Guerry D., Epstein M.N., Greene M.H., Van Horn M. (1984). A study of tumor progression: The precursor lesions of superficial spreading and nodular melanoma. Hum. Pathol..

[2] Jones O.T., Ranmuthu C.K.I., Hall P.N., Funston G., Walter F.M. (2020). Recognising skin cancer in primary care. Adv. Ther..

[3] D’Orazio J., Jarrett S., Amaro-Ortiz A., Scott T. (2013). UV radiation and the skin. Int. J. Mol. Sci..

[4] Qadir M.I. (2016). Skin cancer: Etiology and management. Pak. J. Pharm. Sci..

[5] Gordon R. (2013). Skin cancer: An overview of epidemiology and risk factors. Semin. Oncol. Nurs..

[6] Foote M., Harvey J., Porceddu S., Dickie G., Hewitt S., Colquist S., Zarate D., Poulsen M. (2010). Effect of radiotherapy dose and volume on relapse in Merkel cell cancer of the skin. Int. J. Radiat. Oncol. Biol. Phys..

[7] Jerant A.F., Johnson J.T., Sheridan C.D., Caffrey T.J. (2000). Early detection and treatment of skin cancer. Am. Fam. Physician.

[8] Narayanan D.L., Saladi R.N., Fox J.L. (2010). Ultraviolet radiation and skin cancer. Int. J. Dermatol..

[9] Rodrigues D.D.A., Ivo R.F., Satapathy S.C., Wang S., Hemanth J., Reboucas Filho P.P. (2020). A new approach for classification skin lesion based on transfer learning, deep learning, and IoT system. Pattern Recognit. Lett..

[10] Cai C.J., Winter S., Steiner D., Wilcox L., Terry M. (2019). “Hello AI”: Uncovering the onboarding needs of medical practitioners for human-AI collaborative decision-making. Proc. ACM Hum. Comput. Interact..

[11] Soenksen L.R., Kassis T., Conover S.T., Marti-Fuster B., Birkenfeld J.S., Tucker-Schwartz J., Naseem A., Stavert R.R., Kim C.C., Senna M.M. (2021). Using deep learning for dermatologist-level detection of suspicious pigmented skin lesions from wide-field images. Sci. Transl. Med..

[12] Haggenmüller S., Maron R.C., Hekler A., Utikal J.S., Barata C., Barnhill R.L., Beltraminelli H., Berking C., Betz-Stablein B., Blum A. (2021). Skin cancer classification via convolutional neural networks: Systematic review of studies involving human experts. Eur. J. Cancer.

[13] Allugunti V.R. (2022). A machine learning model for skin disease classification using convolution neural network. Int. J. Comput. Program. Database Manag..

[14] Huang S.-F., Ruey-Feng C., Moon W.K., Lee Y.-H., Dar-Ren C., Suri J.S. (2008). Analysis of tumor vascularity using three-dimensional power Doppler ultrasound images. IEEE Trans. Med. Imaging.

[15] Niu S., Liu Y., Wang J., Song H. (2020). A decade survey of transfer learning (2010–2020). IEEE Trans. Artif. Intell..

[16] Torrey L., Shavlik J. (2010). Transfer learning. Handbook of Research on Machine Learning Applications and Trends: Algorithms, Methods, and Techniques.

[17] Shaha M., Pawar M. Transfer learning for image classification. Proceedings of the 2018 Second International Conference on Electronics, Communication and Aerospace Technology (ICECA).

[18] Krishna S.T., Kalluri H.K. (2019). Deep learning and transfer learning approaches for image classification. Int. J. Recent Technol. Eng. IJRTE.

[19] Hosny K.M., Kassem M.A., Foaud M.M. (2019). Classification of skin lesions using transfer learning and augmentation with Alex-net. PLoS ONE.

[20] Ashraf R., Afzal S., Rehman A.U., Gul S., Baber J., Bakhtyar M., Mehmood I., Song O.-Y., Maqsood M. (2020). Region-of-interest based transfer learning assisted framework for skin cancer detection. IEEE Access.

[21] Fraiwan M., Faouri E. (2022). On the automatic detection and classification of skin cancer using deep transfer learning. Sensors.

[22] Weatheritt J., Rueckert D., Wolz R. Transfer learning for brain segmentation: Pre-task selection and data limitations. Proceedings of the Medical Image Understanding and Analysis: 24th Annual Conference, MIUA 2020.

[23] Lehnert L., Tellex S., Littman M.L. (2017). Advantages and limitations of using successor features for transfer in reinforcement learning. arXiv.

[24] Ganaie M.A., Hu M., Malik A., Tanveer M., Suganthan P. (2022). Ensemble deep learning: A review. Eng. Appl. Artif. Intell..

[25] Yang Y., Lv H., Chen N. (2023). A survey on ensemble learning under the era of deep learning. Artif. Intell. Rev..

[26] Ozkan I.A., Koklu M. (2017). Skin lesion classification using machine learning algorithms. Int. J. Intell. Syst. Appl. Eng..

[27] Singh A., Thakur N., Sharma A. A review of supervised machine learning algorithms. Proceedings of the 2016 3rd International Conference on Computing for Sustainable Global Development (INDIACom).

[28] Sajid P., Rajesh D. (2018). Performance evaluation of classifiers for automatic early detection of skin cancer. J. Adv. Res. Dyn. Control. Syst..

[29] Senan E.M., Jadhav M.E. (2022). Diagnosis of dermoscopy images for the detection of skin lesions using SVM and KNN. Proceedings of the Third International Conference on Sustainable Computing: SUSCOM 2021.

[30] Karamizadeh S., Abdullah S.M., Halimi M., Shayan J., Rajabi M.J. Advantage and drawback of support vector machine functionality. Proceedings of the 2014 International Conference on Computer, Communications, and Control Technology (I4CT).

[31] Lopez A.R., Giro-i-Nieto X., Burdick J., Marques O. Skin lesion classification from dermoscopic images using deep learning techniques. Proceedings of the 2017 13th IASTED International Conference on Biomedical Engineering (BioMed).

[32] Serte S., Demirel H. (2019). Gabor wavelet-based deep learning for skin lesion classification. Comput. Biol. Med..

[33] Mirunalini P., Chandrabose A., Gokul V., Jaisakthi S. (2017). Deep learning for skin lesion classification. arXiv.

[34] Mahbod A., Schaefer G., Wang C., Ecker R., Ellinge I. Skin lesion classification using hybrid deep neural networks. Proceedings of the ICASSP 2019–2019 IEEE International Conference on Acoustics, Speech and Signal Processing (ICASSP).

[35] Younis H., Bhatti M.H., Azeem M. Classification of skin cancer dermoscopy images using transfer learning. Proceedings of the in 2019 15th International Conference on Emerging Technologies (ICET).

[36] Harangi B., Baran A., Hajdu A. Classification of skin lesions using an ensemble of deep neural networks. Proceedings of the 2018 40th Annual International Conference of the IEEE Engineering in Medicine and Biology Society (EMBC).

[37] Shehzad K., Zhenhua T., Shoukat S., Saeed A., Ahmad I., Bhatti S.S., Chelloug S.A. (2023). A Deep-Ensemble-Learning-Based Approach for Skin Cancer Diagnosis. Electronics.

[38] Tschandl P., Rosendahl C., Kittler H. (2018). The HAM10000 dataset, a large collection of multi-source dermatoscopic images of common pigmented skin lesions. Sci. Data.

[39] Ali M.S., Miah M.S., Haque J., Rahman M.M., Islam M.K. (2021). An enhanced technique of skin cancer classification using deep convolutional neural network with transfer learning models. Mach. Learn. Appl..

[40] Weiss K., Khoshgoftaar T.M., Wang D. (2016). A survey of transfer learning. J. Big Data.

[41] Salehi A.W., Khan S., Gupta G., Alabduallah B.I., Almjally A., Alsolai H., Siddiqui T., Mellit A. (2023). A Study of CNN and Transfer Learning in Medical Imaging: Advantages, Challenges, Future Scope. Sustainability.

[42] Ghosal P., Nandanwar L., Kanchan S., Bhadra A., Chakraborty J., Nandi D. Brain tumor classification using ResNet-101 based squeeze and excitation deep neural network. Proceedings of the 2019 Second International Conference on Advanced Computational and Communication Paradigms (ICACCP).

[43] He K., Zhang X., Ren S., Sun J. Deep residual learning for image recognition. Proceedings of the IEEE Conference on Computer Vision and Pattern Recognition.

[44] Howard A.G., Zhu M., Chen B., Kalenichenko D., Wang W., Weyand T., Andreetto M., Adam H. (2017). Mobilenets: Efficient convolutional neural networks for mobile vision applications. arXiv.

[45] Szegedy C., Vanhoucke V., Ioffe S., Shlens J., Wojna Z. Rethinking the inception architecture for computer vision. Proceedings of the IEEE Conference on Computer Vision and Pattern Recognition.

[46] Tan M., Le Q. Efficientnet: Rethinking model scaling for convolutional neural networks. Proceedings of the International Conference on Machine Learning.

[47] Huang G., Liu Z., Van Der Maaten L., Weinberger K.Q. Densely connected convolutional networks. Proceedings of the IEEE Conference on Computer Vision and Pattern Recognition.

[48] Zoph B., Vasudevan V., Shlens J., Le Q.V. Learning transferable architectures for scalable image recognition. Proceedings of the IEEE Conference on Computer Vision and Pattern Recognition.

[49] Kausar N., Hameed A., Sattar M., Ashraf R., Imran A.S., Abidin M.Z.U., Ali A. (2021). Multiclass skin cancer classification using ensemble of fine-tuned deep learning models. Appl. Sci..

[50] Hoang L., Lee S.-H., Lee E.-J., Kwon K.-R. (2022). Multiclass skin lesion classification using a novel lightweight deep learning framework for smart healthcare. Appl. Sci..

[51] Chaturvedi S.S., Tembhurne J.V., Diwan T. (2020). A multi-class skin Cancer classification using deep convolutional neural networks. Multimed. Tools Appl..

[52] Rahi M.M.I., Khan F.T., Mahtab M.T., Ullah A.A., Alam M.G.R., Alam M.A. Detection of skin cancer using deep neural networks. Proceedings of the 2019 IEEE Asia-Pacific Conference on Computer Science and Data Engineering (CSDE).

[53] Sae-Lim W., Wettayaprasit W., Aiyarak P. Convolutional neural networks using MobileNet for skin lesion classification. Proceedings of the 2019 16th International Joint Conference on Computer Science and Software Engineering (JCSSE).

[54] Moldovan D. Transfer learning based method for two-step skin cancer images classification. Proceedings of the 2019 E-Health and Bioengineering Conference (EHB).

[55] Iqbal I., Younus M., Walayat K., Kakar M.U., Ma J. (2021). Automated multi-class classification of skin lesions through deep convolutional neural network with dermoscopic images. Comput. Med. Imaging Graph..

[56] Sai Charan D., Nadipineni H., Sahayam S., Jayaraman U. (2020). Method to Classify Skin Lesions using Dermoscopic images. arXiv.

[57] Phung S.L., Bouzerdoum A., Chai D. (2005). Skin segmentation using color pixel classification: Analysis and comparison. IEEE Trans. Pattern Anal. Mach. Intell..

[58] Shrivastava V.K., Londhe N.D., Sonawane R.S., Suri J.S. (2017). A novel and robust Bayesian approach for segmentation of psoriasis lesions and its risk stratification. Comput. Methods Programs Biomed..

[59] Oliveira R.B., Mercedes Filho E., Ma Z., Papa J.P., Pereira A.S., Tavares J.M.R. (2016). Computational methods for the image segmentation of pigmented skin lesions: A review. Comput. Methods Programs Biomed..

[60] Sumithra R., Suhil M., Guru D. (2015). Segmentation and classification of skin lesions for disease diagnosis. Procedia Comput. Sci..

[61] Liu K., Suri J.S. (2005). Automatic Vessel Indentification for Angiographic Screening. U.S. Patent.

[62] Li H., He X., Zhou F., Yu Z., Ni D., Chen S., Wang T., Lei B. (2018). Dense deconvolutional network for skin lesion segmentation. IEEE J. Biomed. Health Inform..

[63] Kamnitsas K., Ledig C., Newcombe V., Simpson J., Kane A., Menon D., Rueckert D., Glocker B. (2017). Efficient multi-scale 3D CNN with fully connected CRF for accurate brain lesion segmentation. Med. Image Anal..

[64] El-Baz A., Gimel’farb G., Suri J.S. (2015). Stochastic Modeling for Medical Image Analysis.

[65] Suri J.S. (2001). Two-dimensional fast magnetic resonance brain segmentation. IEEE Eng. Med. Biol. Mag..

[66] Acharya U.R., Faust O., Alvin A.P., Krishnamurthi G., Seabra J.C., Sanches J., Suri J.S. (2013). Understanding symptomatology of atherosclerotic plaque by image-based tissue characterization. Comput. Methods Programs Biomed..

[67] Acharya U.R., Sree S.V., Krishnan M.M.R., Krishnananda N., Ranjan S., Umesh P., Suri J.S. (2013). Automated classification of patients with coronary artery disease using grayscale features from left ventricle echocardiographic images. Comput. Methods Programs Biomed..

[68] Acharya U.R., Faust O., Sree S.V., Molinari F., Saba L., Nicolaides A., Suri J.S. (2011). An accurate and generalized approach to plaque characterization in 346 carotid ultrasound scans. IEEE Trans. Instrum. Meas..

[69] Singh J., Singh N., Fouda M.M., Saba L., Suri J.S. (2023). Attention-Enabled Ensemble Deep Learning Models and Their Validation for Depression Detection: A Domain Adoption Paradigm. Diagnostics.

[70] Suri J.S., Agarwal S., Pathak R., Ketireddy V., Columbu M., Saba L., Gupta S.K., Faa G., Singh I.M., Turk M. (2021). COVLIAS 1.0: Lung segmentation in COVID-19 computed tomography scans using hybrid deep learning artificial intelligence models. Diagnostics.

[71] Suri J.S., Agarwal S., Chabert G.L., Carriero A., Paschè A., Danna P.S.C., Saba L., Mehmedović A., Faa G., Singh I.M. (2022). COVLIAS 2.0-cXAI: Cloud-based explainable deep learning system for COVID-19 lesion localization in computed tomography scans. Diagnostics.

[72] Saba L., Banchhor S.K., Suri H.S., Londhe N.D., Araki T., Ikeda N., Viskovic K., Shafique S., Laird J.R., Gupta A. (2016). Accurate cloud-based smart IMT measurement, its validation and stroke risk stratification in carotid ultrasound: A web-based point-of-care tool for multicenter clinical trial. Comput. Biol. Med..

[73] Shrivastava V.K., Londhe N.D., Sonawane R.S., Suri J.S. (2016). Computer-aided diagnosis of psoriasis skin images with HOS, texture and color features: A first comparative study of its kind. Comput. Methods Programs Biomed..

[74] Molinari F., Mantovani A., Deandrea M., Limone P., Garberoglio R., Suri J.S. (2010). Characterization of single thyroid nodules by contrast-enhanced 3-D ultrasound. Ultrasound Med. Biol..

[75] Saba L., Banchhor S.K., Araki T., Viskovic K., Londhe N.D., Laird J.R., Suri H.S., Suri J.S. (2018). Intra-and inter-operator reproducibility of automated cloud-based carotid lumen diameter ultrasound measurement. Indian Heart J..

[76] Acharya U.R., Mookiah M.R.K., Sree S.V., Yanti R., Martis R.J., Saba L., Molinari F., Guerriero S., Suri J.S. (2014). Evolutionary algorithm-based classifier parameter tuning for automatic ovarian cancer tissue characterization and classification. Ultraschall Med. Eur. J. Ultrasound.

[77] Agarwal M., Agarwal S., Saba L., Chabert G.L., Gupta S., Carriero A., Pasche A., Danna P., Mehmedovic A., Faa G. (2022). Eight pruning deep learning models for low storage and high-speed COVID-19 computed tomography lung segmentation and heatmap-based lesion localization: A multicenter study using COVLIAS 2.0. Comput. Biol. Med..

[78] Suri J.S., Agarwal S., Gupta S.K., Puvvula A., Viskovic K., Suri N., Alizad A., El-Baz A., Saba L., Fatemi M. (2021). Systematic review of artificial intelligence in acute respiratory distress syndrome for COVID-19 lung patients: A biomedical imaging perspective. IEEE J. Biomed. Health Inform..

[79] Suri J.S., Bhagawati M., Agarwal S., Paul S., Pandey A., Gupta S.K., Saba L., Paraskevas K.I., Khanna N.N., Laird J.R. (2022). UNet Deep Learning Architecture for Segmentation of Vascular and Non-Vascular Images: A Microscopic Look at UNet Components Buffered With Pruning, Explainable Artificial Intelligence, and Bias. IEEE Access.

[80] Suri J.S., Agarwal S., Jena B., Saxena S., El-Baz A., Agarwal V., Kalra M.K., Saba L., Viskovic K., Fatemi M. (2022). Five strategies for bias estimation in artificial intelligence-based hybrid deep learning for acute respiratory distress syndrome COVID-19 lung infected patients using AP (ai) Bias 2.0: A systematic review. IEEE Trans. Instrum. Meas..

[81] Vaswani A., Shazeer N., Parmar N., Uszkoreit J., Jones L., Gomez A.N., Kaiser L., Polosukhin I. Attention is all you need. In Proceedings of the 31st Conference on Neural Information Processing Systems (NIPS 2017).

[82] Khan S., Naseer M., Hayat M., Zamir S.W., Khan F.S., Shah M. (2022). Transformers in vision: A survey. ACM Comput. Surv. CSUR.

[83] Suri J., Liu K., Singh S., Laxminarayan S., Zeng X., Reden L. (2002). Shape recovery algorithms using level sets in 2-D/3-D medical imagery: A state-of-the-art review. IEEE Trans. Inf. Technol. Biomed..

